# Effects of *Rhodomyrtus tomentosa* Leaf Extract on Staphylococcal Adhesion and Invasion in Bovine Udder Epidermal Tissue Model

**DOI:** 10.3390/nu7105410

**Published:** 2015-10-15

**Authors:** Auemphon Mordmuang, Shiv Shankar, Usa Chethanond, Supayang Piyawan Voravuthikunchai

**Affiliations:** 1 Department of Microbiology and Excellent Research Laboratory on Natural Products, Faculty of Science and Natural Product Research Center of Excellence, Prince of Songkla University, Hat Yai, Songkhla 90112, Thailand; mordmuang.ap@hotmail.com (A.M.); shivbiotech@gmail.com (S.S.); 2 Faculty of Veterinary Science, Prince of Songkla University, Hat Yai, Songkhla 90112, Thailand; usa.ch@psu.ac.th

**Keywords:** bacterial adhesion, bovine mastitis, cell surface hydrophobicity, *Rhodomyrtus tomentosa*, staphylococci

## Abstract

Bovine mastitis is one of the most important infectious diseases in dairy herds, and staphylococci are the most important etiologic agents of this disease. Antibiotics and chemical agents used in livestock for prevention and cure of the disease can accumulate in milk and give rise to food safety concerns. *Rhodomyrtus tomentosa* leaf extract was studied as an alternative approach to reduce the bacterial infections. The ethanolic extract of this plant demonstrated antibacterial activity with minimum inhibitory concentration (MIC) values as low as 16–64 μg/mL against staphylococcal isolates. In addition, the extract had an effect on the bacterial cell surface properties by increasing its hydrophobicity in a concentration dependent manner. To further extend the antibacterial efficacy, silver nanoparticles synthesized with the extract, a pure rhodomyrtone, and liposomal encapsulated rhodomyrtone were applied and their inhibitory effects on bacterial adhesion and invasion were determined by *ex vivo* study in a bovine udder epidermal tissue model. These agents exerted remarkable antibacterial activity against staphylococci and decreased the adhesion of the bacterial cells to the tissues. These results supported that *R. tomentosa* ethanolic extract could be applied as an alternative agent for bovine udder care in dairy farms.

## 1. Introduction

Bovine mastitis is one of the main food safety concerns in dairy industries worldwide. The concern was considered to be an important issue because of the risk of bacterial contamination and chemical residue in milk, mainly due to the use of antibiotics and disinfectants to control the disease. *Staphylococcus* spp. such as *Staphylococcus aureus* are the most frequent harmful pathogens that causes of mastitis and results in considerable economic losses to dairy farmers [1]. The interaction between *S. aureus* and bovine mammary epithelial tissues is considered to play an important role in the pathogenesis of the disease. The adhesion and invasion mechanisms of *S. aureus* are generally assumed to be essential for bacterial colonization and internalization. The bacteria can produce many virulence factors such as beta-toxin, endotoxin, catalase, and other virulence factors that facilitate bacterial invasion and intracellular replication inside the host cells [2]. The persistence of the bacteria inside bovine mammary glands or epithelial cells can help the bacteria to evade normal host immune systems [3]. Moreover, production of staphylococcal enterotoxins in raw milk during mastitis episodes may subsequently provide a high risk to consumers [4].

In dairy herds, staphylococcal bovine mastitis is a contagious disease and it is easily *transmitted from cow to cow during milking. The bacteria can survive both inside and outside of the udders, or on the teat skin and it is usually spread because of deficiencies in proper milking hygiene*. Consequently, the raw milk can carry this harmful pathogen as well as its virulence toxins. In most cases antibiotics and disinfectants are usually prescribed for bovine udder treatment. The bulk milk can be contaminated with the residues of drugs or chemical agents during their extensive use. Therefore, it may lead to resistance development of antibiotic resistance in the pathogen [5] or allergic reactions in humans [6].

*Rhodomyrtus tomentosa* (Aiton) Hassk is a small shrub belonging to the Myrtaceae family. It is one of the herbal plants that is commonly used in traditional medicine in Southeast Asia. This plant has been used to treat diarrhoea [7], and for wound healing [8]. Recently, the plant has been commercially used for treatment of urinary tract infections [9]. This plant species has been reported to possess antibacterial activity against a number of foodborne pathogenic bacteria such as *S. aureus* [10], *Enterococcus faecalis*, *Bacillus subtili*, and *B. cereus* [11,12]. Moreover, phenolic compounds, flavonoids, and tannins from the plant extract have been reported to possess antioxidant [13] and anti-inflammatory properties [14]. Rhodomyrtone, a pure compound, one of the chemical constituents isolated from this plant, possesses strong antibacterial activity against *S. aureus* which is closed to the activity of vancomycin [15] and can enhance the expression of local host immunity against *S. aureus* infections [16].

Therefore, the objectives of the present research were to study the effects of *R. tomentosa* extract on staphylococcal cell surfaces and bacterial adhesion and invasion through *ex vivo* study using the bovine udder epidermal tissue model. The use of various agents, including rhodomyrtone, silver nanoparticles, and liposomal mediated delivery system were studied for their antibacterial activity and inhibitory effects against bacterial adhesion and invasion, in order to improve the potential use of this plant extract for treating bovine mastitis.

## 2. Experimental Section

### 2.1. Bacterial Isolates and Culture

Staphylococcal isolates were obtained from dairy herds kept in farms located in Phatthalung province, Thailand. The bacterial isolates were identified based on their biochemical characteristics, such as mannitol (*MSA*, Merck, Darmstadt, Germany) fermentation, catalase reaction, and coagulase production. Representative bovine mastitis coagulase-positive staphylococci No. 31 (BMPOS No. 31) (Phatthalung Dairy Cattle Cooperative, Phattalung, Thailand) and bovine mastitis coagulase-negative staphylococci No. 12 (BMNEG No. 12) (Phatthalung Dairy Cattle Cooperative, Phattalung, Thailand) were used to assess their cell surface hydrophobicity and ability to invade the bovine udder tissue model. *Staphylococcus aureus* ATCC 29213 and *S. epidermidis* ATCC 35984 were included as standard strains. All isolates were cultured on tryptic soy agar (TSA, *Difco*, Bordeaux, *France*) at 37 °C for 18–24 h and maintained in Tryptic soy broth (TSB, *Difco*, Bordeaux, *France*) at 37 °C for 3–5 h. The bacterial suspensions were adjusted for optical density (OD) of 0.1 at 600 nm (10^8^ colony forming units (CFU)/mL) and further diluted with sterile saline solution (8.5% NaCl) to obtain a cell suspension containing 1 × 10^6^ CFU/mL for the antibacterial assay.

### 2.2. Preparation of R. tomentosa Ethanolic Extract and Rhodomyrtone

*R. tomentosa* leaves were dried in an oven at 60 °C for 48 h and ground in an electric blender. Dried leaf powder was extracted with 95% ethanol at room temperature for seven days. The extract was evaporated using a rotary evaporator (BUCHI Rotavapor R-114, Büchai Labortechnik AG, Flawil, Swizerland) until it was completely dry then dissolved in 10% dimethyl sulphoxide (DMSO, Sigma, Darmstadt, Germany) before use. Pure rhodomyrtone, isolated from this plant, was dissolved in 100% DMSO before use.

### 2.3. Synthesis of Silver Nanoparticles from R. tomentosa Ethanolic Extract

The green synthesis of silver nanoparticles (AgNPs) was performed as previously described [17]. Briefly, an aqueous solution of 1 mM silver nitrate (AgNO_3_) was reacted with 0.01% (*w*/*v*) of *R. tomentosa* ethanolic extract at a final concentration for synthesis. The mixture was kept in the dark at 28 °C with 150 rpm for 48 h in a rotary shaker operating at 150 rpm for 48 h. Bioreduction of Ag^+^ ion in the reaction medium was measured by UV-visible spectrophotometry (Perkins Elmer LAMBDA 25 UV/Vis spectrophotometer, Waltham, MA, USA) in the wavelengths of 200–800 nm. The mixture without AgNO_3_ or the ethanolic extract itself was used as controls in the synthesis. Silver nanoparticles solutions were purified by centrifugation at 14,500 rpm for 1 h and the pellet was resuspended in sterile distilled water. Non-reduced AgNO_3_ ions and unbound extract residues were removed by repeated centrifugation steps 3 times, as above. The amount of the extract was estimated by spectrophotometry using its absorbance at 670 nm. The collected pellet was resuspended in sterile distilled water and checked for sterility before use.

### 2.4. Preparation of Liposomal Encapsulated Rhodomyrtone

Liposomal encapsulated rhodomyrtone was prepared by the ethanol injection method as previously described [18]. The lipid phase was made from a mixture of soybean phosphatidylcholine (Sigma, St. Louis, MO, USA) and cholesterol from lanolin (Fluka, Tokyo, Japan) at a ratio of 4:1 in 10 mL of absolute ethanol. Ten microliters of a rhodomyrtone solution (100 mg/mL) was dissolved in absolute ethanol and used as a water phase mixture. The suspensions of lipid and water phases were sonicated for 30 min and further warmed to 60 °C in a water bath separately The water phase was added to the lipid phase immediately and continuously mixed for 5 min. Ethanol was then removed using a rotary evaporator (Eyela Rotary Vacuum Evaporator N-100 series, Tokyo, Japan). Subsequently, the liposomal encapsulated rhodomyrtone was kept at room temperature until used.

### 2.5. Determination of Minimum Inhibitory Concentration (MIC) and Minimum Bactericidal Concentration (MBC)

*R. tomentosa* ethanolic extract (REE), rhodomyrtone, synthesized AgNPs from the ethanolic extract (AgNPs-REE), and liposomal encapsulated rhodomyrtone were used as antimicrobial agents in this experiment. The minimum inhibitory concentration (MIC) of each antimicrobial agent was determined by a broth microdilution method according to performance standards for antimicrobial susceptibility testing [19]. Briefly, twenty microliters of the agents were separately added and diluted by two-fold serial dilutions in 96-well microtitre plates. The total volume was made up to 100 μL by adding 80 μL of Mueller Hinton broth (MHB, Difco, Bordeaux, France). One hundred microliters of bacterial suspension (10^6^ CFU/mL) were inoculated into each well containing the antimicrobial agents and incubated at 37 °C for 18 h. Blank liposome, 1% DMSO (Sigma, Darmstadt, Germany), and AgNPs without capping the extract (AgNPs-WR) were included as controls. Vancomycin was used as the standard antimicrobial agent in this experiment.

MBC was determined subsequently to the MIC assay. A 100 μL aliquot from the wells around the MIC value was seeded onto TSA plates. The plates were observed for bacterial growth after incubation at 37 °C for 18 h. The experiment was carried out in triplicate.

### 2.6. Microbial Adhesion to Hydrocarbon (MATH) Test

The cell-surface hydrophobicity of staphylococcus was measured by the MATH test [20,21]. Staphylococcal isolates were maintained in TSB medium supplemented with 8 to 256 μg/mL of *R. tomentosa* ethanolic extract at 37 °C for 3–5 h. After incubation, the bacterial cells were collected by centrifugation at 4000 *g* for 5 min, washed twice with sterile saline solution, and the cell density adjusted to OD 0.3 at 600 nm (OD initial). The bacterial cells incubated without the extract were used as a control. Three mL of the cell suspension was placed in a glass tube and 0.25 mL of toluene was added. The mixtures were thoroughly mixed for 2 min by vortex and allowed to equilibrate at room temperature for 10 min. After the toluene phase had separated from the culture phase, the OD of the aqueous phase (OD final) was determined at 600 nm by spectrophotometry. The hydrophobicity index (%) was calculated as:

(1) $$\frac{\text{O}.\text{D}.\text{ initial }-\text{ O}.\text{D}.\text{ final }}{\text{O}.\text{D}.\text{ initial}}\times 100$$

Staphylococcal isolate with a hydrophobic index greater than 70% was classified as being hydrophobic.

### 2.7. Ex Vivo Anti-Adhesion Assay

This *ex vivo* experiment was prepared by a modified excision-based sampling method as previously described [22,23]. Bovine udder epidermal tissue was obtained from freshly slaughtered dairy cows. The tissues were cut in sterile condition to produce approximately 1 gram and trimmed into a strip with a surface area of 10 mm^2^ and a depth of about 3 mm. Each strip was washed with distilled water to remove dust or debris and decontaminated with 70% ethyl alcohol for 10 min. The tissue strips were washed thoroughly with distilled water and used immediately. The bacterial isolates were subcultured in TSB at 37 °C for 4 h and the cell density adjusted to 10^8^ CFU/mL. The bacterial cells were suspended in pH 7.0, phosphate buffered saline (PBS) containing 1/8×, 1/4×, 1/2×, and 1 × MIC of the antimicrobial agents and incubated at 37 °C for various incubation periods (at 0, 1, 4, and 8 h). The test culture containing 1% DMSO was used as a control/untreated.

The transfection was performed by transferring the tissue strip into a pre-treated bacterial culture and co-incubation at 37 °C for 1–2 h. Non-attached bacteria were removed by washing the tissues with sterile PBS. The tissue was placed in an individual sterile stomacher bag containing 9 mL of phosphate buffer and homogenized using the stomacher machine (BagMixer^®^ 400 lab blender, Interscience, St. Nom, France) for 120 s on a normal setting. An aliquot from the homogenized solution was collected and the amount of adherent bacteria was measured by viable cell counting on TSA and MSA. The experiment was carried out in triplicate.

### 2.8. Ex Vivo Anti-Infection Assay

The bovine udder epidermal tissue was prepared as described for the *ex vivo* anti-adhesion assay. The tissue strips were co-incubated with a bacterial suspension (10^8^ CFU/mL) at 37 °C for 4 h. After transfection, non-attached bacteria were removed by washing the tissues with sterile PBS. The seeded tissue was treated with 1×, 2×, 4×, and 8× the MIC value of the antimicrobial agents and incubated at 37 °C for various incubation periods (at 0, 2, 4, 8, 16, 20, and 24 h). A culture containing 1% DMSO was used as a control/untreated.

After treatment, each tissue was picked from the culture and transferred to an individual stomacher bag containing 9 mL of phosphate buffer. Mechanical mashing by the stomacher machine as described above was used to extrude the invasive bacteria inside the tissue. Bacterial numbers were determined by viable cell counting on TSA and MSA plates. The experiment was carried out in triplicate.

### 2.9. Statistical Analysis

The difference in the number of adherent bacteria or internalized bacteria between the controls and each treatment after the enumeration of CFU by viable plate count was assessed for significance (* *p* < 0.05) using one-way ANOVA in SPSS software version 13 for windows (SPSS Inc., Chaicago, IL, USA). All values are expressed as the means ± standard deviation (SD).

## 3. Results

### 3.1. Antibacterial Activity

The REE possessed strong antibacterial activity against all tested staphylococcal isolates with MIC and MBC values that ranged from 16–64 μg/mL and 32–128 μg/mL, respectively (Table 1). Rhodomyrtone itself had a profound antibacterial activity with MIC and MBC values that ranged from 0.5–1 μg/mL and 1–2 μg/mL, respectively, which is close to the activity of vancomycin. Moreover, the silver nanoparticles (AgNPs) synthesised from *R. tomentosa* ethanolic extract and the liposomes containing rhodomyrtone were used as antimicrobial agents against staphylococci. The synthesized AgNPs with capped REE (AgNPs-REE) exhibited MIC and MBC values that ranged from 4–8 μg/mL and 8–32 μg/mL, respectively. AgNPs-WR had a lower activity with higher MIC and MBC values, that ranged from 128–512 μg/mL and >800 μg/mL, respectively, while the liposomal encapsulated rhodomyrtone had MIC and MBC values that ranged from 2–4 μg/mL and 8–32 μg/mL, respectively. Liposomes not containing rhodomyrtone had no antibacterial activity (Table 1).

**Table 1:** Minimum inhibitory concentrations (MIC) and minimum bactericidal concentrations (MBC) of antimicrobial agents against staphylococcal isolates.

Antimicrobial Agents	MIC/MBC (μg/mL)			
	*S. aureus* ATCC 29213	*S. epidermidis* ATCC 35984	BMPOS No. 31	BMNEG No. 12
*R. tomentosa* ethanolic extract	32/64	16/32	64/128	32/64
Rhodomyrtone	0.5/1	0.5/1	1/2	0.5/1
AgNPs-REE	4/32	8/16	8/32	4/8
AgNPs-WR	512/>800	256/>800	512/>800	128/>800
Liposomal encapsulated rhodomyrtone	2/16	2/8	4/32	2/16
Liposome	NA	NA	NA	NA
Vancomycin	1/2	0.5/1	1/2	0.5/1

NA; not applicable; AgNPs-REE, synthesized AgNPs with capped *R. tomentosa* ethanolic extract; AgNPs-WR, AgNPs without the extract.

### 3.2. Effects of R. tomentosa Ethanolic Extract on Staphylococcal Cell Surface Hydrophobicity

**Figure 1:**
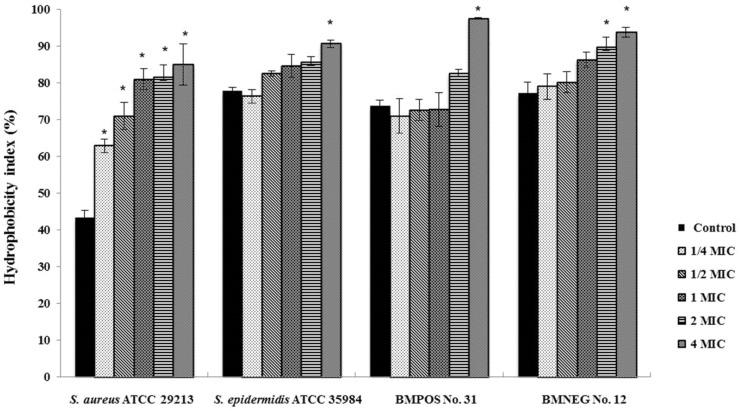
Effects of *R. tomentosa* ethanolic extract on staphylococcal cell surface hydrophobicity. Hydrophobicity index was quantified by an assay for microbial adhesion to hydrocarbons (MATH) after treating the bacterial cell with 1/4 × minimum inhibitory concentration (MIC), 1/2 × MIC, 1 × MIC, 2 × MIC, and 4 × MIC of the extract. The natural hydrophobicity of the bacterial cells was observed in a control/untreated. The mean value ± standard deviation (SD) from at least duplicates are illustrated. * *p* < 0.05 demonstrated a significant difference between the tests and the control.

The effects of *R. tomentosa* ethanolic extract on the surface properties of staphylococcal cells were determined as it had been hypothesized that the extract may modify the hydrophobicity or aggregation activity of the bacteria. The two clinical isolates obtained from the bovine teat canals possessed a higher level of cell surface hydrophobicity than *S. aureus* reference strain. These bacteria were classified as hydrophobic bacteria with a hydrophobicity index of more than 70%. It might result from bacterial survival and the infective abilities of the bacteria inside the bovine teat canal and udder tissues. A high biofilm producing strain, *S. epidermidis* ATCC 35984 demonstrated the nature of cell surface hydrophobicity that was higher than the other tested staphylococci as expected. Furthermore, the cells treated with the ethanolic extract demonstrated a higher level of hydrophobicity than the untreated cells. The ethanolic extract modified the bacterial cell surface by increasing the hydrophobicity in a concentration-dependent manner. However, the hydrophobicity of all tested isolates, except for *S. aureus* ATCC 29213, was not significantly increased by exposure to sub-inhibitory concentrations of the extract (Figure 1).

### 3.3. Anti-Adhesion Activity

**Figure 2:**
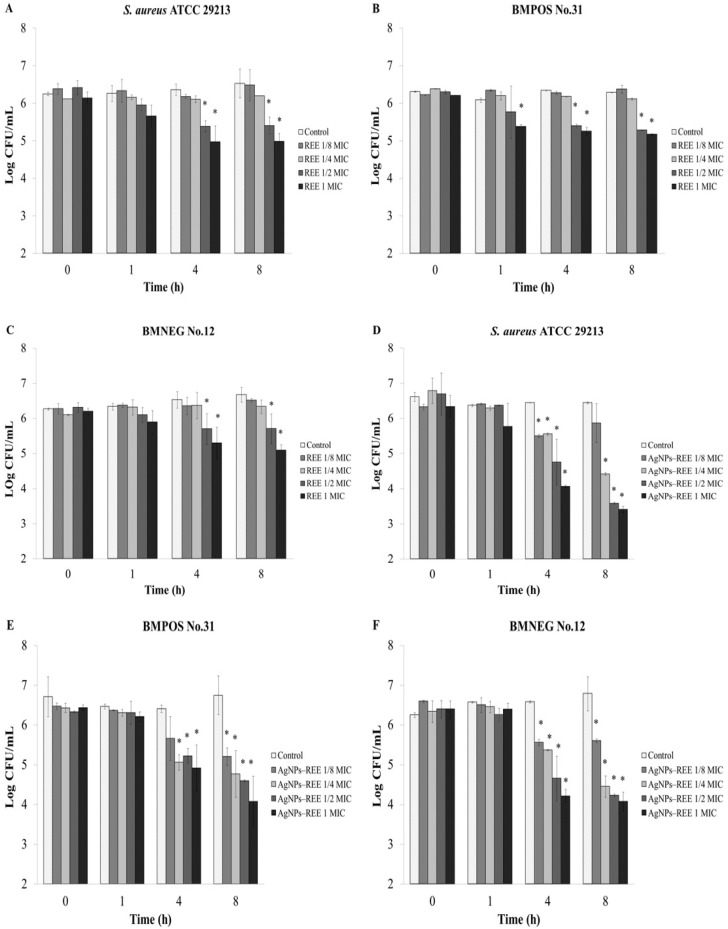
Quantification of bacterial adherence by *ex vivo* anti-adhesion assay. Each bar represents the number of adherent bacteria to bovine udder tissue evaluated by viable plate count. Bacterial isolates were treated with 1/8 ×minimum inhibitory concentration (MIC), 1/4 × MIC, 1/2 × MIC, and 1 × MIC of the ethanolic extract; *R. tomentosa* ethanolic extract (REE) (**A**–**C**) or synthesized AgNPs with capped REE (AgNPs–REE) from the ethanolic extract (**D**–**F**) at various time points (h). 1% DMSO was used as a control. The data represent the pooled results from a least two experiments and are expressed as a mean value ± standard deviation (SD). * *p* < 0.05, compared to the control.

**Figure 3:**
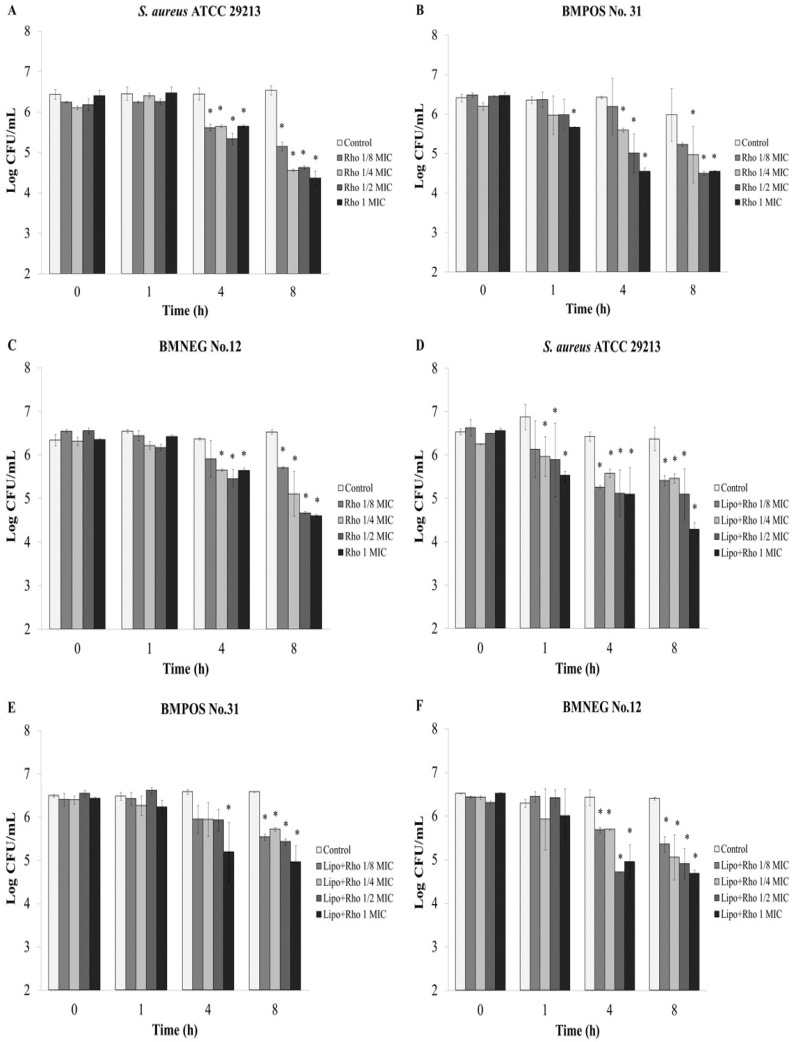
Quantification of bacterial adherence by *ex vivo* anti-adhesion assay. Each bar represents the number of adherent bacteria to the bovine udder tissue evaluated by a viable plate count. Bacterial isolates were treated with 1/8 × minimum inhibitory concentration (MIC), 1/4 × MIC, 1/2 × MIC, and 1 × MIC of rhodomyrtone; Rho (**A**–**C**) or liposome encapsulated rhodomyrtone; Lipo + Rho (**D**–**F**) at various time points (h). 1% DMSO was used as a control. The data represent the pooled results from two experiments and are expressed as a mean value ± standard deviation (SD). * *p* < 0.05, compared to the control.

An *ex vivo* experiment was designed to assess the effects of the extract on the adhesion ability of the staphylococci to the udder epidermal tissue. The extract at half the MIC and 1 × MIC can reduce adherent bacteria after treatment for 4 h with a significant difference when compared with the control (*p* < 0.05) (Figure 2A–C). Moreover, the number of adherent bacterial cells were decreased after 1 h treatment with 1 × MIC of the extract (Figure 2B). The synthesized AgNPs-REE with concentrations that ranged from 1/8 to 1 × MIC had strong activity against the adherence of bacteria to the tissue strip for all tested isolates after treatment for 4 h (Figure 2D–F). Rhodomyrtone, as the pure compound isolated from *R. tomentosa* extract also exhibited an inhibitory activity on bacterial adhesion to the bovine udder epidermal tissue. The number of adherent bacterial cells were significantly decreased after 4 h of treatment with 1/8 to 1 × MIC of the compound (Figure 3A–C). The compound with a concentration of 1 × MIC significantly reduced the number of BMPOS No.31 adherent cells after treatment for 1 h (Figure 3B). Furthermore, the total adherent *S. aureus* ATCC 29213 and BMNEG NO.12 bacterial cells were significantly decreased after treatment with 1/8 to 1 × MIC of liposome encapsulated rhodomyrtone for 4 h (Figure 3D,F), whereas the number of BMPOS NO.31 cells were significant decreased after treatment with 1 × MIC (Figure 3E).

### 3.4. Anti-Invasion Activity

**Figure 4:**
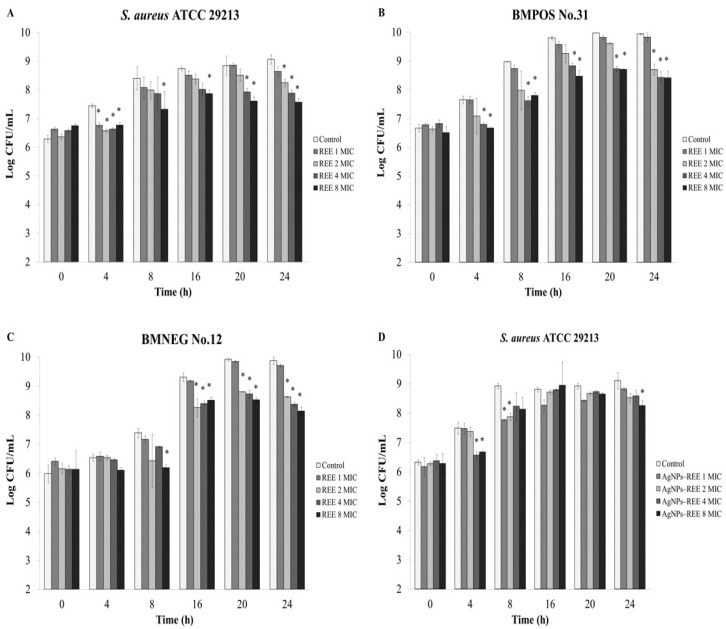

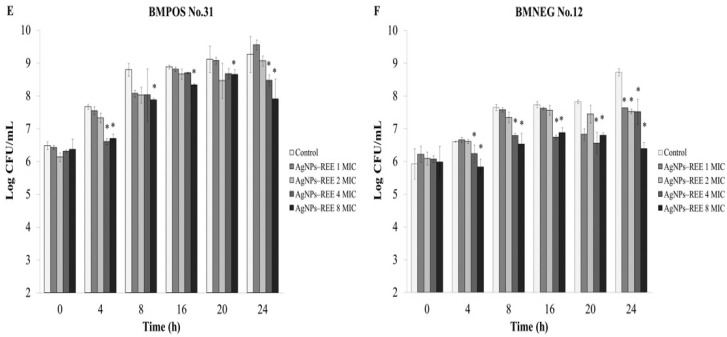
Quantification of bacterial invasion by *ex vivo* anti-infection assay. Each bar represents the number of invasive bacteria isolated from the bovine udder tissue as evaluated by the viable plate count. The bacterial transfected tissues were treated with 1 × minimum inhibitory concentration (MIC), 2 × MIC, 4 × MIC, and 8 × MIC of the ethanolic extract; *R. tomentosa* ethanolic extract (REE) (**A**–**C**) or synthesized AgNPs with capped REE (AgNPs-REE) from the ethanolic extract (**D**–**F**) at various time points (h). 1% DMSO was used as a control. The data represent the pooled results from two experiments and are expressed as a mean value ± standard deviation (SD). * *p* < 0.05, compared to the control.

**Figure 5:**
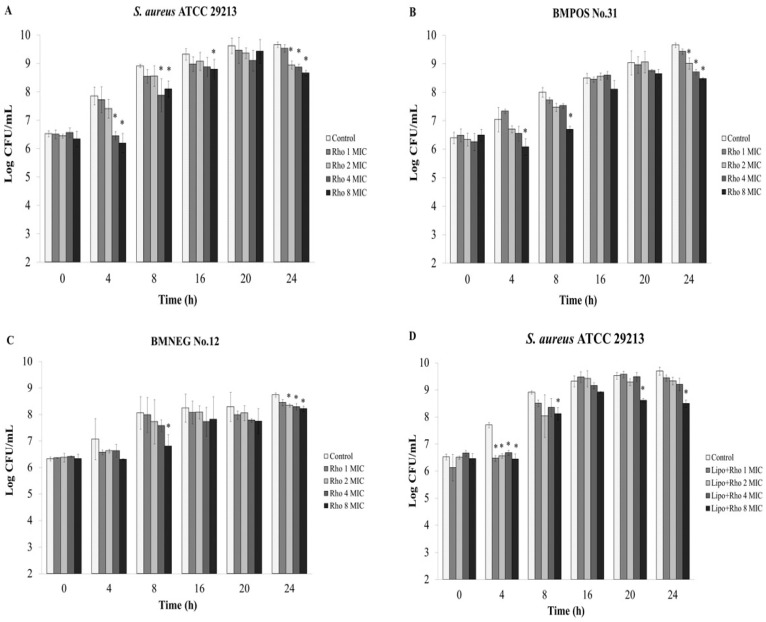

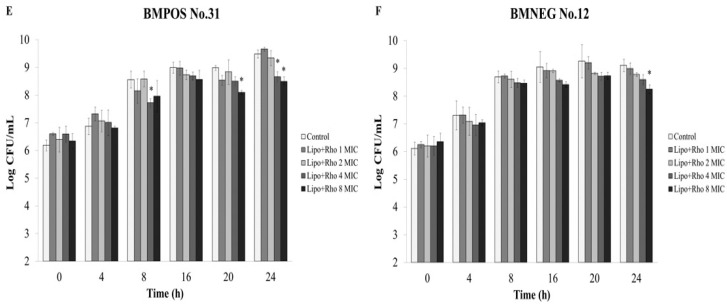
Quantification of the bacterial invasion using *ex vivo* anti-infection assay. Each bar represents the number of invasive bacteria in the bovine udder tissue evaluated by a viable plate count. The bacterial transfected tissues were treated with 1 × minimum inhibitory concentration (MIC), 2 × MIC, 4 × MIC, and 8 × MIC of rhodomrtone; Rho (**A**–**C**) or liposome encapsulated rhodomyrtone; Lipo + Rho (**D**–**F**) at various time points (h). 1% DMSO was used as a control. The data represent the pooled results from two experiments and are expressed as a mean value ± standard deviation (SD). * *p* < 0.05, compared to the control.

*Ex vivo* experiment was carried out to evaluate the number of invasive or internalized bacteria inside the udder epidermal tissue after treatment with the *R. tomentosa* ethanolic extract. The extract exhibited an anti-invasion activity in a concentration dependent manner (Figure 4A–C). The extract at 4 × MIC and 8 × MIC inhibited bacterial invasion by the tested staphylococcal isolates after treatment for 4 h with significant differences when compared to a control, while for BMNEG No.12 there was no significant difference (*p* < 0.05). The number of internalized bacterial cells in the tissue strips were decreased by increasing concentrations of the extract after treatment for 16 h (Figure 4A–C). In a similar way, the synthesized AgNPs-REE treatments for 8–24 h decreased the numbers of internalized bacteria with increasing concentrations of the nanoparticles (Figure 4D–F). The concentrations of 4 × MIC and 8 × MIC of synthesized AgNPs-REE reduced the number of internalized bacteria of all tested isolates after recovery from the bovine udder tissue at 4 h. The numbers of BMPOS No.31 and *S. aureus* ATCC 29213 were significantly decreased after treatment with 8 × MIC of the synthesized AgNPs–REE at all time points (Figure 4E–F). Furthermore, the recovered internalized bacterial cells from the different concentrations of rhodomyrtone were nearly the same as those of the liposomal encapsulated rhodomyrtone treatments during the incubation period (Figure 5). The number of internalized bacterial cells in the bovine tissue for each treatment increased with increasing recovery times that were seen respectively after continuous incubation for 24 h. This might be due to bacterial survival and intracellular replication inside the tissue matrices after invasion into the bovine udder epidermal tissues.

## 4. Discussion

Many attempts have been made to establish alternative ways to avoid the use of drugs or chemicals to treat mastitis in dairy herds. Plants are sources of potentially useful natural substances for the development of new antimicrobial products. They have become more attractive for organic dairy farm management and have been studied extensively as therapeutic agents. In this study the potential antibacterial activities of an ethanolic extract of a Thai traditional herbal plant, *R. tomentosa* against staphylococci that cause mastitis was evaluated. The extract had MIC and MBC values that ranged from 16–64 μg/mL and 32–128 μg/mL, respectively. In comparison to other research work on natural compounds, an extract from the hop cone *Humulus lupulus* L. (Cannabaceae) demonstrated antibacterial activity against *S. aureus* strains with MIC values that ranged from 31.2–125 μg/mL [24]. Methanolic extracts from *Cenchrus ciliaris* and *Coccinia grandis* showed antibacterial activity against *S. aureus* that caused mastitis with MIC values of 125 μg/mL [25]. In addition, salvipisone, a diterpenoid compound isolated from the hairy roots of *Salvia sclarea* displayed bactericidal activity against *S. aureus* and *S. epidermidis* strains with MIC values that ranged from 9.37–18.75 μg/mL [26]. Berberine isolated from *Coptidis rhizoma* exerted a bacteriostatic effect on *S. epidermidis starins* with MIC and MBC values that ranged from 64–256 μg/mL and 256–1024 μg/mL, respectively [27].

The ethanolic extract from *R. tomentosa* at supra-inhibitory concentrations affected bacterial cells by increasing their surface hydrophobicity in a concentration dependent manner. The extract affected the bacterial hydrophobicity but there was no correlation with its antibacterial activity according to the MIC and MBC studies. An increase in the cell surface hydrophobicity could render microorganisms more readily susceptible to phagocytosis. Loss of both the K and O antigens increased the surface hydrophobicity and susceptibility of *Klebsiella aerogenes* to phagocytosis [28]. We speculated that the composition of the plant extract did influence the bacterial cell surface by modifications to its hydrophobic characteristics. Protein A, lipoteichoic acid, capsular polysaccharide, fibronectins, and adhesin proteins that are present on the bacterial cell wall and their cytoplasmic membrane were considered to be important factors involved in the hydrophobicity and changes to the staphylococci cell surface [29,30]. Furthermore, some of the membrane-related proteins have been recognized as essential components for the adhesion and invasion mechanisms of staphylococci. The ethanolic extract might have an ability to modify these structural proteins on the bacterial cell surface. Therefore, interactions between the bacteria and the bovine epithelial tissues may be influenced by nonspecific surface charges of the bacterial cell surface. According to the effect of the chemical constituents of the ethanolic extract, an earlier study using transcriptomic analysis reported that rhodomyrtone induced a significant modulation of gene expression involved in the biosynthesis of amino acids and peptidoglycan by *S. aureus* [31]. Results from a proteomic analysis on methicillin-resistant *S. aureus* indicated that this compound might have an effect on the proteins associated with carbohydrate metabolism, cell wall biosynthesis, and other proteins involved in protein degradation, oxidative stress, a putative hydrolase, and conserved hypothetical proteins, as well as the cell-surface antigens and virulence factors that were also inhibited after treatment [32]. Other chemical constituents including flavonoids, phenolic compounds, triterpenoids, and tannins in addition to rhodomyrtone have been obtained in extracts from *R. tomentosa* [33,34]. The presence of these components in the plant extract would have similar effects to those isolated from cranberry ethanolic extracts that had an inhibitory effect on the cell wall biosynthesis of *S. aureus* [35].

Therefore, in this study the effects of the *R. tomentosa* extracts and rhodomyrtone itself on the adhesion and invasive ability of staphylococci was determined using bovine udder epidermal tissue as an *ex vivo* model. The extract at its MIC value significantly reduced the number of BMPOS No.31 adherent bacteria within 1 h while at a half × MIC the extract significantly reduced the number of adherent bacteria within 4 h for all tested isolates. Rhodomyrtone itself also demonstrated an inhibitory activity on staphylococcal adhesion to bovine udder epidermal tissue. The extract with concentrations that ranged from of 1/8–1 × MIC significantly reduced the number of adherent bacteria within 4 h. AgNPs induced a very high research interest in biomedical science due to their antimicrobial properties. A green method for the synthesis of AgNPs has advantages over a chemical reduction and physical processes in being environmentally friendly and cost effective [17]. Therefore, in the present work, an ethanolic extract of *R. tomentosa* was used to synthesise AgNPs-REE by a green method. The synthesized AgNPs-REE showed antibacterial activity with MIC and MBC values that ranged from 4–8 μg/mL and 8–32 μg/mL, respectively. The AgNPs synthesized with an *R. tomentosa* acetone extract (RAE) demonstrated antibacterial activity against *S. aureus* with MIC and MBC values in the range of 3.1–6.2 μg/mL and 6.2–50 μg/mL, respectively, and was also dependent on the temperature and RAE concentration used [17]. In another study, synthesized gold, silver, and gold-silver alloy nanoparticles from *Lansium domesticum* fruit peel extract demonstrated antibacterial activity and biocompatible activity using *in vitro* studies [36]. The AgNPs synthesized by *L. domesticum* extract showed MIC/MBC of 16/32 μg/mL and 8/16 μg/mL against *S. aureus* and *E. coli*, respectively. Moreover, AgNPs synthesized by *L. domesticum* showed no cytotoxicity against C2C12 cell lines up to a 40 μg/mL concentration. These studies showed that the concentration of AgNPs for antibacterial activity was much lower than the concentration that exhibited the cytotoxic effects [17,36]. Therefore, the combined effect of AgNPs and *R. tomentosa* ethanolic extract would be a promising alternative to reduce microbial contamination. Liposomes containing rhodomyrtone showed MIC and MBC values that ranged from 2–4 μg/mL and 8–32 μg/mL, respectively. These two formulations were shown to have a profound activity against bacterial adherence for all the tested isolates after treatment for 4 h with lower concentrations of 1/8 × MIC, as expected. The result indicated that the anti-adhesion effects of the synthesized AgNPs-REE were not correlated to its antibacterial activity in the method used to determine the MIC and MBC values.

An *ex vivo* anti-invasion assay was further performed to observe the therapeutic efficacy of the ethanolic extract, rhodomyrtone, AgNPs-REE, and the liposomal formulation, since in this study these agents demonstrated an inhibitory activity on staphylococcal adhesion. The results indicated that the ethanolic extract exhibited an anti-invasion activity in a concentration dependent manner. However, the numbers of internalized bacteria increased after continuous incubation. This might be because the large molecule of the extract could not easily penetrate into the complex tissue matrices for killing the intracellular bacteria. Therefore, to solve a problem about drug diffusion, rhodomyrtone and synthesized AgNPs-REE that have smaller molecular sizes were also used in this experiment. The numbers of internalized bacterial cells significantly decreased after treatment with 2–8 × MIC of rhodomyrtone for 24 h in a concentration dependent manner. The synthesized AgNPs-REE reduced the numbers of internalized bacteria after treatment for 4–24 h in a concentration dependent manner, but it was not a significant reduction. Moreover, the liposome delivery system was applied to the liposomal encapsulated rhodomyrtone formulation, in order to enhance the diffusion barrier properties of the compound for penetration through lipid-rich tissues. However, the numbers of recovered invasive bacteria from the rhodomyrtone treatments at each concentration were nearly the same as those recovered from the treatments with the liposomal encapsulated rhodomyrtone. The results indicated that the polarity or the charge interactions between the antibacterial compound and the mammalian cells might influence the penetration capacity of the antibacterial compound into the tissue matrix. In addition the numbers of internalized bacteria increased over time after continuous incubation for 24 h. That might be due to bacterial invasion and replication inside the epithelial cells during the incubation period.

## 5. Conclusions

In conclusion, we have provided evidence for an antibacterial activity, for modifications to bacterial cell surface hydrophobicity, and for an anti-adhesion effect by an ethanolic *R. tomentosa* extract and pure rhodomyrtone, on staphylococcal induced bovine mastitis. The results indicate that the *R. tomentosa* extract and its various formulations could be applied for use as an alternative method to reduce *Staphylococcus* infections from contaminants in dairy farms.
